# Lysosomal positioning diseases: beyond substrate storage

**DOI:** 10.1098/rsob.220155

**Published:** 2022-10-26

**Authors:** Gianluca Scerra, Valeria De Pasquale, Melania Scarcella, Maria Gabriella Caporaso, Luigi Michele Pavone, Massimo D'Agostino

**Affiliations:** ^1^ Department of Molecular Medicine and Medical Biotechnologies, University of Naples Federico II, Via Sergio Pansini 5, 80131 Naples, Italy; ^2^ Department of Veterinary Medicine and Animal Productions, University of Naples Federico II, Via Federico Delpino 1, 80137 Naples, Italy

**Keywords:** lysosome, microtubule tracks, membrane contact sites, positioning, trafficking, lysosomal storage diseases

## Abstract

Lysosomal storage diseases (LSDs) comprise a group of inherited monogenic disorders characterized by lysosomal dysfunctions due to undegraded substrate accumulation. They are caused by a deficiency in specific lysosomal hydrolases involved in cellular catabolism, or non-enzymatic proteins essential for normal lysosomal functions. In LSDs, the lack of degradation of the accumulated substrate and its lysosomal storage impairs lysosome functions resulting in the perturbation of cellular homeostasis and, in turn, the damage of multiple organ systems. A substantial number of studies on the pathogenesis of LSDs has highlighted how the accumulation of lysosomal substrates is only the first event of a cascade of processes including the accumulation of secondary metabolites and the impairment of cellular trafficking, cell signalling, autophagic flux, mitochondria functionality and calcium homeostasis, that significantly contribute to the onset and progression of these diseases. Emerging studies on lysosomal biology have described the fundamental roles of these organelles in a variety of physiological functions and pathological conditions beyond their canonical activity in cellular waste clearance. Here, we discuss recent advances in the knowledge of cellular and molecular mechanisms linking lysosomal positioning and trafficking to LSDs.

## Graphical abstract

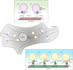

## I. Introduction

Lysosomal storage diseases (LSDs) are genetic disorders that collectively affect 1 : 5000 live births [1]*.* LSDs are in most cases inherited as autosomal recessive traits and their common features include a broad variability in presentation, including severe, early-onset forms (infancy and childhood) that can lead to premature death, adult-onset forms with attenuated phenotypes, and a high degree of clinical heterogeneity associated with age at onset, severity of symptoms, extent of central nervous system involvement and disease progression. Multiplex dysostosis, hepatosplenomegaly, angiokeratomas, facial dimorphisms, corneal opacities and vision impairment, muscle deficits and skeletal changes, immune defects, recurrent infections, seizures, cardiovascular disorders, psychomotor developmental delay, and cognitive decline are the most common clinical manifestations in LSDs that may have a devastating impact on the quality of life of both patients and their families [2–12].

LSDs are characterized by intra-lysosomal accumulation of metabolic products in multiple tissues and organs, as a result of mutations in genes encoding for proteins that are critical for lysosomal functions, including lysosomal enzymes, lysosomal integral membrane proteins and proteins involved in post-translational modification and trafficking of lysosomal proteins. Any deficiency of these proteins may lead to the dysfunction of multiple cellular processes such as lysosomal pH regulation, endocytosis, autophagy, exocytosis and Ca^2+^ homeostasis [10,13–20]. While in the past decades, the pathophysiology of LSDs has been considered only the result of defective substrate degradation due to the lack of lysosomal enzymes, more recent evidence demonstrates the complexity of the molecular mechanisms involved in the pathogenesis of these diseases. However, it has yet to be fully elucidated. Regardless, significant advances towards the understanding of lysosomal biology have provided novel and important insights into the type of cellular perturbations occurring in LSDs and their effects, thus paving the way for the development of new therapeutic approaches for the treatment of LSDs [21–29]. Currently, lysosomes are not merely considered terminal degradative organelles, but key metabolic hubs involved in nutrient sensing, secretion, gene regulation, plasma membrane (PM) repair, metal ion homeostasis, lipid transport and other fundamental cellular processes [30–35]. For most of these processes, the lysosomal function is strictly dependent on the correct positioning and motility of the compartment, both controlled by complex mechanisms involving microtubule- and actin-based motors, protein complexes, and membrane contact sites (MCSs) between organelles in response to nutrient levels and lipid distribution in membranes [36–46]*.* Therefore, the deregulation of mechanisms underlying lysosomal positioning, motion through the cytoplasm and fusion with specific compartments to receive and deliver substrates for further processing may be responsible for the dysfunctions associated with various diseases including LSDs as well as cancer and neurodegenerative disorders [13,23,26,47–49]. In this review, we discuss recent findings on spatially compartmentalized mechanisms regulating the distribution and dynamics of endolysosomal organelles and highlight the contribution of these mechanisms to the pathogenesis of LSDs.

## 2. Protein complexes move the endolysosomes back and forth alongside the microtubule tracks

The endolysosomal compartment consists of multiple copies of single membrane vesicles containing numerous luminal hydrolases responsible for the degradation of a wide range of substrates including sugars, lipids, proteins and nucleic acids [50,51]. Lysosomal hydrolases need an acidic pH to correctly work and guarantee proper organelle function [33]. The acidic pH of the lysosomal lumen is controlled by the V-ATPase, a large vacuolar channel that pumps protons across the lysosomal membrane and inside the lumen [52]. Lysosomes can degrade substrates of both intracellular and extracellular origin. Extracellular macromolecules reach the lysosomes through endocytosis [53], and together with cytoplasmic macromolecules, damaged proteins and old organelles are processed through the autophagic pathway [54,55]. Thanks to its movement on microtubule tracks, the endolysosomal compartment is very dynamic, and its positioning within each cell type determines its correct function.

**Figure 1.**
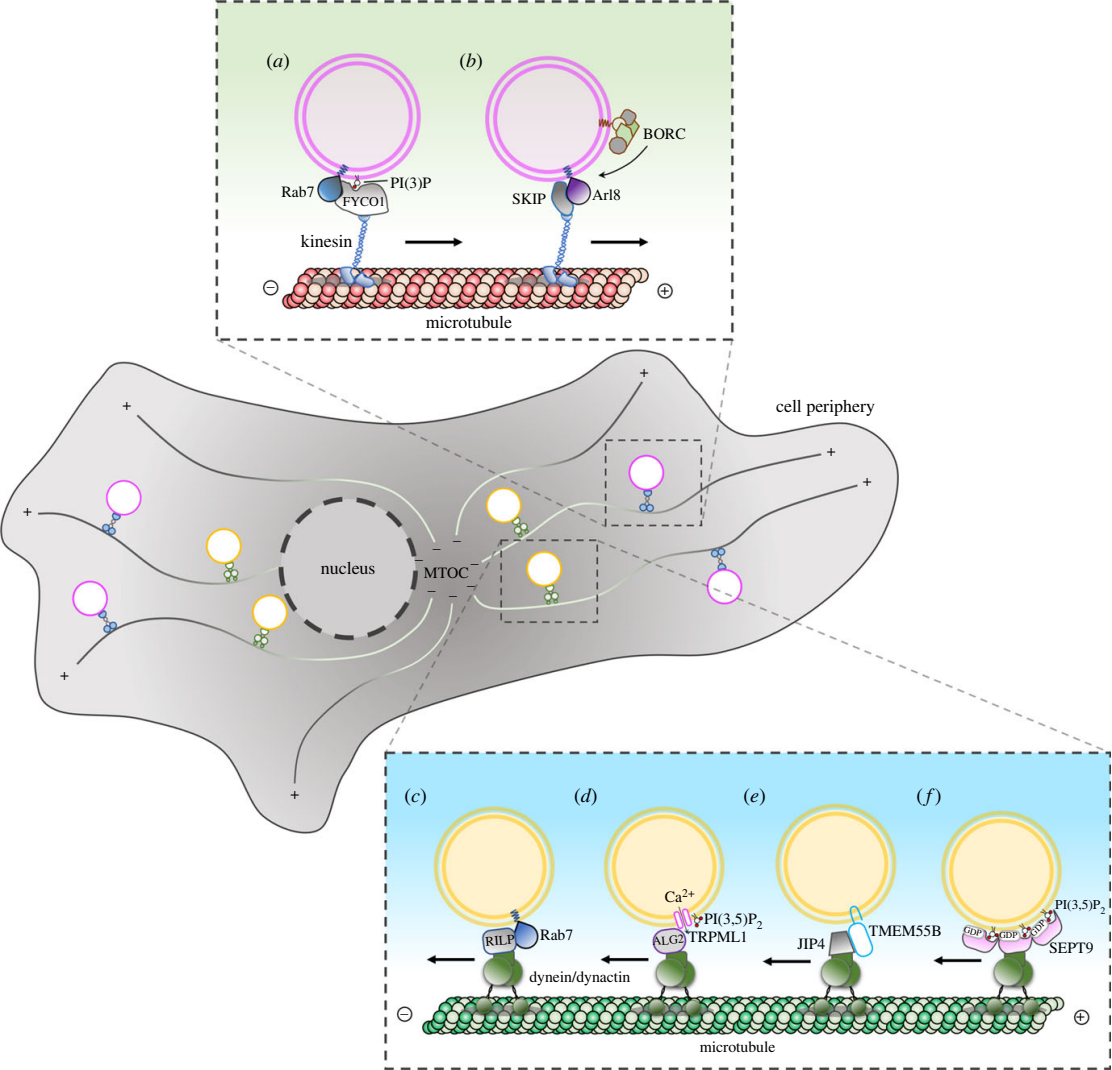
The bidirectional movement of endolysosomes on microtubule tracks is driven by different macromolecular complexes. (*a*) The kinesin adaptor FYCO1 interacts with active Rab7 and PI(3)P on the lysosomal membrane to recruit kinesin-1 to the lysosomal surface. (*b*) The lysosomal multi-subunit complex BORC allows the recruitment of the small GTPase Arl8 to lysosomes. Arl8 recruits the effector protein SKIP to bind kinesin-1 motor protein. (*c*) The small GTPase Rab7 recruits the effector protein RILP and the dynein–dynactin motor on the lysosomal surface. (*d*) The role of Ca^2+^ in endolysosomal positioning. High levels of PI(3,5)P2 on endolysosomal membranes stimulate the opening of the TRPML1 channel to promote Ca^2+^ efflux which allows the recruitment of calcium sensor ALG2 at the endolysosomal membranes. Then, ALG2 recruits the dynein–dynactin complex to TRPML1-containing lysosomes. (*e*) Under nutrients or cholesterol depletion conditions, the lysosomal transmembrane protein TMEM55B is upregulated and promotes interaction with dynein through the adaptor protein JIP4. (*f*) High levels of PI(3,5)P2 on endolysosomal membranes promote the recruitment of oligomeric GDP-bound form of SEPT9 which mediates the binding to dynein–dynactin complex.

Endolysosomal organelles move bidirectionally between the centre and the periphery of a cell alongside microtubule tracks (figure 1). In non-polarized cells, microtubules are radially distributed with their minus-ends at a perinuclear microtubule-organizing centre (MTOC) and their plus-ends pointing towards the cell periphery. On the contrary, polarized cells such as epithelial cells and neurons show more complex microtubule organizations, with some microtubules pointing their plus-ends towards the perinuclear area. As such, in these specialized cells, centrifugal or centripetal transport depends on specific microtubules to which the organelles are attached. Most kinesin motors drive organelle transport from the minus-end towards the plus-end (anterograde or centrifugal transport) [56], while the dynein motor drives organelle transport in the other direction (retrograde or centripetal transport). The coupling of endolysosomal organelles to kinesins is often mediated by small GTPases and their effectors, as well as membrane phospholipids, which function as organelle cargo adaptors. For instance, the small GTPase Rab7, a Ras-related protein, can bind the effector protein FYVE And Coiled-Coil Domain Autophagy Adaptor 1 (FYCO1) which, in combination with phosphatidylinositol 3-phosphate PI(3)P, recruits the kinesin-1 motor to drive the plus-end transport of endolysosomes towards the cell periphery [57] (figure 1*a*). On the other hand, the recruitment of Kinesin Family Member 5B (KIF5B) and KIF1A/KIF1Bb to the late endosomes (LEs)/lysosomes depends on the multi-subunit complex BLOC-one-related complex (BORC) and the small GTPase Arl8 (ADP Ribosylation Factor Like GTPase 8) [58–60] (figure 1*b*). In this case, the association of the same organelle with distinct kinesins drives movement in different regions of the cell (KIF5B towards the periphery and KIF1A/KIF1Bb towards MTOC) [61].

In contrast with the variety of kinesins, there is only one cytoplasmic dynein heavy chain protein [62]. It associates with the multi-subunit complex dynactin and promotes the lysosomal transport towards the microtubule minus-ends. Many different factors, including small GTPases, adaptor proteins, phosphoinositides and cation channels, modulate dynein-dependent centripetal transport of endolysosomes in a different fashion (figure 1*c–f*) [63–73]. Emerging evidence has demonstrated how the centripetal localization of the endolysosomal compartment is controlled by septins (SEPTs) [74,75], a family of GTP-binding proteins, which multi-merize into higher order oligomers and polymers that associate with cell membranes and cytoskeleton [76]. In the endocytic pathway, SEPTs interact preferentially with endolysosomes enriched with PI(3,5)P2 and Rab7 [77]. However, unlike the Rab7 GTPase and lysosomal membrane proteins, which recruit dynein through cytoplasmic adaptor proteins such as Rab Interacting Lysosomal Protein (RILP), C-Jun-amino-terminal kinase-interacting protein 4 (JIP4) and ALG4 (Phosphomannomutase), SEPT9 is a membrane-associated GTPase that directly interacts with dynein. Under oxidative stress, SEPT9 associates preferentially with dynein in its GDP-bound state, which favours SEPT9 dimerization and assembly into higher ordered oligomers. As such, in contrast with the monomeric small GTPases of the Rab and Arf families, which are activated by GTP, SEPT9 provides a GDP-activated platform to directly recruit multiple dynein–dynactin complexes [76] (figure 1*f*).

The role of small GTPases in moving endolysosomes has been also revealed in cancer cells. Here, lysosomes relocate at the cell periphery near the PM leading to excessive lysosomal secretion [47,78] accompanied by the release of lysosomal proteases, such as cathepsins. In the extracellular environment, these proteases compromise the integrity of the extracellular matrix (ECM), thereby facilitating tumour growth and invasion [14,79–84]. Such a peripheral distribution is controlled by the relative concentration of Rab7 and Arl8b, with peripheral lysosomes containing more Arl8b and less Rab7 [43]. Therefore, during cancer progression, changes in the expression levels of Rab GTPases can influence the preferential movement of the lysosomes towards the cell periphery to stimulate their exocytosis [67,85].

## 3. Endoplasmic reticulum controls endolysosomal positioning via membrane contact sites

**Figure 2.**
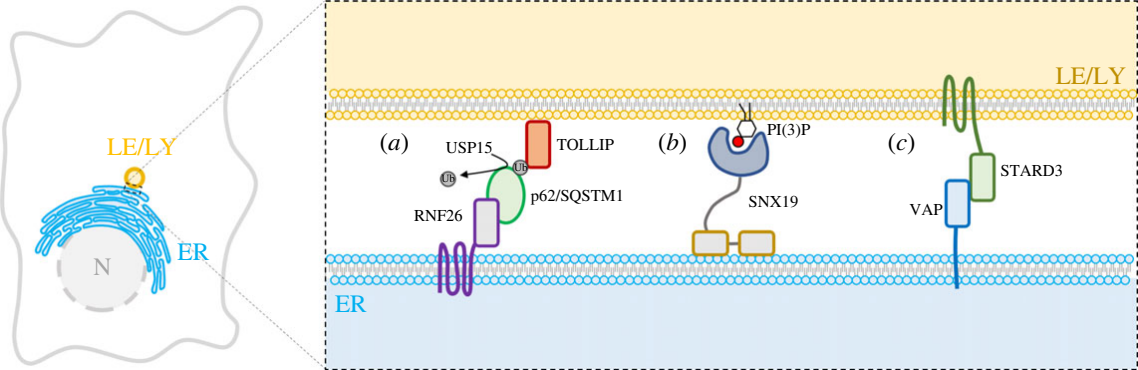
MCSs between endoplasmic reticulum and endolysosomes. The schematic view of different protein complexes involved in the establishment of MCSs between the ER and the endolysosomal compartment (LE/LY). (*a*) The ER-resident transmembrane protein RNF26 recruits and promotes the ubiquitination of the adaptor protein p62/SQSTM1 which interacts with the membrane-bound endolysosomal protein TOLLIP. These MCSs are reverted by the de-ubiquitinating enzyme USP15. (*b*) Sortin nexin-19 (SNX19) interacts with PI(3)P-enriched membranes to promote contacts between the ER and LE/LY membranes. (*c*) The lysosomal transmembrane protein STARD3 establishes interaction with the vesicle-associated ER membrane protein VAP.

The overall distribution of endolysosomal compartment within the cell is not only determined by the interaction and activity of motor proteins on microtubules but also by the establishment of physical contact with other organelles, such as the endoplasmic reticulum (ER) network. Although the endolysosomal compartment can move bidirectionally between centre and periphery of each cell, at a steady state, it is largely concentrated around the MTOC. Accordingly, this distribution is controlled by the E3 ubiquitin-protein ligase RNF26, which localizes at the level of the perinuclear ER [86]. In this location, RNF26 interacts and promotes the ubiquitination of the adaptor protein p62/SQSTM1 which, in turn, interacts with various endolysosomal adaptor proteins, including the Toll-interacting protein TOLLIP, localized on the LEs and phagosomes, epidermal growth factor receptor substrate 15 EPS15, localized on early endosomes, and Tax1-binding protein 1 (TAX1BP1), localized at the trans-Golgi network. These adaptor proteins are characterized by the presence of specific ubiquitin- and membrane-binding domains that allow tethering of their respective compartments to the perinuclear area of the ER where RNF26 resides. These interactions are reverted by the de-ubiquitinating enzyme Ubiquitin-Specific Peptidase 15 (USP15), which releases organelles from the ER, allowing their movement on microtubules through kinesin and dynein motors [86] (figure 2*a*).

As such, the free diffusion of cargo molecules is restricted at stable MCSs, suggesting that this mechanism could play a role in cargo sorting. A recent work has shown that endolysosomes, whose size was expanded by the accumulation of intraluminal substrates, establish extended contact sites with the ER membrane through vesicle-associated membrane proteins VAP which strongly limit endolysosome motility and secretion [22], thereby confirming the need for transient interactions to drive endolysosomal motility, size control and function [87]. Accordingly, it has been recently demonstrated that also in neurons the ER establishes extended MCSs with enlarged and less motile lysosomes [36]. Such interaction occurs in the pre-axonal region, the soma of the neuron, where kinesin 1, bound to the ER protein P180, provides the pulling force for driving lysosome fission and the subsequent axonal transport of the lysosomes towards the cell periphery. Furthermore, another recent work has demonstrated that the sorting nexin protein SNX19 tethers endolysosomes to the ER membranes and decreases their motility, contributing to their localization at the MTOC area [88]. This tethering depends on two N-terminal transmembrane domains that anchor SNX19 to the ER, and a phox homology domain that binds PI(3)P on the endolysosomal membrane (figure 2*b*). Thus, these studies have identified a fundamental mechanism controlling the shape, motility and positioning of endolysosomes that involves their tethering to the ER network.

Another important feature of the dynamic formation of MCSs between endolysosomes and ER is linked to the control of the fission and fusion processes, key cellular mechanisms for organelle trafficking and function. For instance, it has been proposed that fission of early endosomes [89] and LEs occurs at the contact sites with the ER [90]. Although the molecular mechanism of this process remains to be elucidated, some evidence suggests that the ER contributes to the budding of retromer-containing tubules from the endosomes [70]. This mechanism involves the same ER proteins that participate in the control of dynein and kinesin binding to LEs such as VAP-A and its paralogue VAP-B. These proteins establish interactions with the PI(4)P transporter oxysterol-binding protein (OSBP) and retromer-associated SNX2 protein which lead to actin nucleation and consequent budding of retromer-containing tubular membranes. Like OSBP, two other endolysosomal lipid transfer proteins, StAR-Related Lipid Transfer Domain-Containing 3 (STARD3) and STARD3 N-Terminal Like (STARD3NL), interact with VAP proteins via their FFAT motif, thereby providing another way for the ER to contact endolysosomes [91] (figure 2*c*). The fusion of LE/lysosomes with other organelles, such as autophagosomes, is also subject to regulation by the interaction of ORP1L (member of OSBP family) with VAP-A at the ER membranes [92,93]. The release of ORP1L from VAP-A is required for the formation of the Rab7-RILP-PLEKHM1 complex which, in turn, recruits the multi-meric tethering complex HOPS on the LE/lysosome membranes to drive the membrane fusion between LEs/lysosomes and autophagosomes [93,94]. Altogether, these works illustrate how the motility of the endolysosomal compartment is integrated with fission and fusion mechanisms, highlighting the vital role of the ER network in regulating these processes.

## 4. Nutrient availability controls the positioning of the lysosomes

The mammalian target of rapamycin (mTOR), initially identified in *Saccharomyces cerevisiae* cells treated with rapamycin, which promotes irreversible cell cycle arrest [95], forms the core of two different multi-protein complexes, mTOR complex 1 (mTORC1) and mTOR complex 2 (mTORC2), which differ for their accessory proteins [96,97]. Whereas mTORC2 regulates cell survival, metabolism and cytoskeletal structure [98], mTORC1 functions as a central regulator of metabolism, thus ensuring that cells only grow under favourable conditions [99]. mTOR signalling is dependent on its serine/threonine kinase activity towards target substrates. mTORC1 complex triggers cell proliferation and cell growth by stimulating anabolism and suppressing catabolism through the phosphorylation of key effector proteins such as the ribosomal protein S6 kinase (S6K) and the eukaryotic translation initiation factor 4E-binding protein (4E-BP) [99,100]. Indeed, mTORC1 complex regulates cell growth by either promoting anabolism, including ribosome biogenesis as well as nucleotide and lipid synthesis, or inhibiting catabolic processes like autophagy. mTORC1 consists of the core protein mTOR in complex with the regulatory-associated protein of mTOR (Raptor), mammalian lethal with SEC13 protein 8 (mLST8), proline-rich Akt substrate of 40 kDa (PRAS40) and DEP domain-containing mTOR interacting protein (Deptor). This macromolecular complex is recruited to the lysosomes when nutrients, including amino acids, glucose and cholesterol, are abundant. Different amino acids can activate mTORC1 through distinct signalling cascades and mechanisms [101]. In particular, 10 amino acids, namely alanine, arginine, asparagine, glutamine, histidine, leucine, methionine, serine, threonine and valine, stimulate mTORC1 activity, although with different kinetics. For instance, leucine, arginine and methionine potently activate mTORC1, promoting S6K1 phosphorylation very rapidly (approx. 15 min), whereas glutamine acts more slowly (approx. 60 min) [102,103].

**Figure 3.**
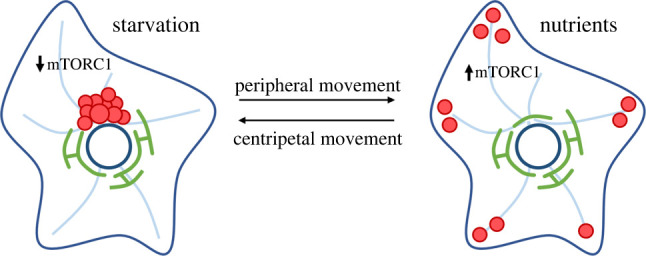
Nutrients influence lysosome positioning. Under starvation, e.g. in the absence of amino acids and growth factors, mTORC1 signalling is reduced and lysosomes are moved at the cell centre towards MTOC by a dynein–dynactin motor complex. On the contrary, nutrient availability stimulates mTORC1 signalling and promotes peripheral lysosomal localization thanks to the action of kinesin-based motor proteins.

Intracellular distribution of the endolysosomal compartment is tightly linked to nutrient availability (figure 3). For instance, nutrient starvation not only inhibits mTORC1 activity but also stimulates endolysosomal movement towards the perinuclear region of the cell [46]. Additionally, nutrient withdrawal induces translocation of MiT/TFE family transcription factors, such as TFEB and TFE3, to the nucleus to promote lysosomal biogenesis and autophagosome formation [73,104].

Autophagy starts from the de novo formation of double-membrane structures called phagophores and proceeds through the sequestration of cytosolic components such as macromolecules and organelles in the expanding phagophores to form double-membrane vesicles called autophagosomes. The maturation of autophagosomes into autolysosomes may occur by two distinct routes: the fusion of autophagosomes first with LEs to form amphisomes and subsequently with lysosomes to form autolysosomes, or directly with lysosomes. The autolysosomal cargo is then degraded by acidic lysosomal hydrolases, and the breakdown products are exported via lysosomal transporters for recycling [105]. Thus, the degradation of endosomal and autophagosomal material needs the fusion of these last two compartments with lysosomes, forming endolysosomes [106–108] and autolysosomes [109–111], respectively. Once the material is degraded by the lysosomal hydrolyses, the fission machinery drives the lysosomal reformation [112,113]. Indeed, under physiological conditions, endolysosomes and autolysosomes are transient organelles. Overall, autophagy plays a critical role especially in neuronal functions and survival, avoiding a too high concentration of toxic proteins and contributing to the degradation of aged or damaged organelles, such as mitochondria [114]*.* During autophagy, vesicles containing damaged proteins and cell debris are transported to the minus-end of microtubules where they fuse with lysosomes responsible for the degradation of their contents. The promotion of autophagy links the inhibition of mTORC1 activity, during starvation, to the activation of catabolic processes via lysosomal activity [73,104]. Since amino acid and nutrient starvation is reversible, the minus-end transport of lysosomes can be rapidly switched towards the cell periphery by supplying back nutrients and amino acids [46]. Amino acid availability quickly activates mTORC1, which phosphorylates TFEB and TFE3, preventing their translocation into the nucleus and inhibiting lysosomal biogenesis [34,115,116]. Therefore, nutrient availability controls lysosome positioning and biogenesis via mTORC1 signalling and TFEB, TFE3 transcription factors.

## 5. Impairment of endolysosomal positioning and trafficking in lysosomal storage diseases

**Figure 4.**
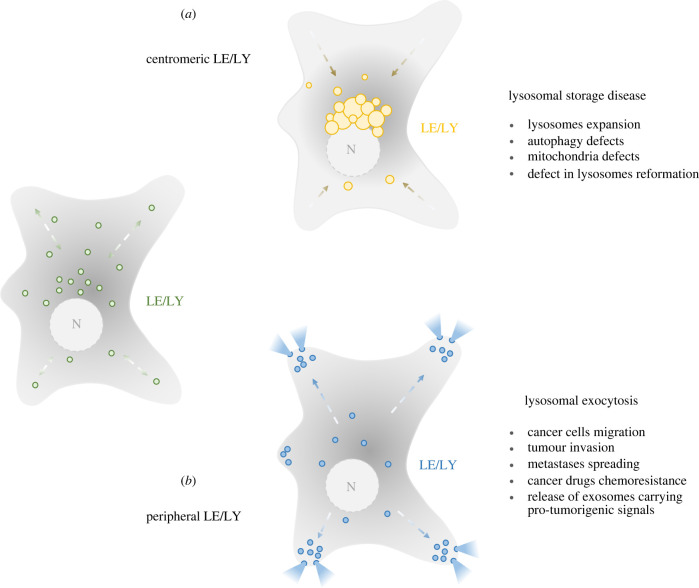
Correlation between lysosome positioning and pathological defects. (*a*) Centripetal/centromeric localization of endolysosomes is often seen in pathological conditions such as lysosomal storage diseases characterized by multiple cellular defects including the centripetal clustering of enlarged lysosomes, defects in the autophagic pathways, mitochondrial activity impairment and lysosomal reformation defects. (*b*) Peripheral lysosomes are generally referred to as secretory lysosomes which have relevant implications for tumour progression and drug chemoresistance.

Thanks to its bidirectional movement on microtubule tracks, the endolysosomal compartment can occupy two opposite physiological locations such as the centre or the periphery of a cell (figure 4*a*). However, despite the physiological relevance, peculiar endolysosomal positioning is often linked to pathological conditions too [117–119]. For instance, centripetal localization of lysosomes is often found in LSDs, which are characterized by a clustering of enlarged substrate-accumulating endolysosomes at the MTOC area (figure 4*b*) [8,21,22,120]. By contrast, the peripheral localization of the endolysosomes (figure 4*c*), which is typically associated with lysosomal exocytosis, is often considered a cancer-related factor [47,121–124].

In LSDs, partially degraded substrates accumulate within lysosomes due to the deficiency of lysosomal hydrolases, transporters, accessory proteins or membrane proteins. Although lysosomal proteins are ubiquitously distributed, the accumulation of undegraded materials in LSD patients is normally restricted to those cells, tissues and organs where substrate turnover is high. Accumulation of the primary storage material can cause the accumulation of secondary substrates such as glycosphingolipids, phospholipids and cholesterol, which triggers a chain of alterations in other biochemical and cellular functions, resulting in the severe pathology of LSDs [27] (table 1).

**Table 1.** Most common LSDs with their defective enzymes, accumulated substrates and altered lysosome-related pathways.

LSD	defective protein (gene)	stored substrate(s)	altered lysosomal pathway(s)	references
aspartylglucosaminuria	aspartylglucosaminidase (AGA)	glycoasparagines	lysosomal enzyme deficiency	[125]
Danon	lysosome-associated membrane protein 2 (LAMP2)	glycogen	intracytoplasmic trafficking autophagy block	[126]
				[127]
Fabry	α-galactosidase (GLA)	globotriaosylceramide (Gb3)	autophagy-lysosomal pathway endocytosis and lysosomal degradation of endothelial calcium-activated intermediate-conductance potassium ion channel 3.1 (KCa3.1)	[128,129]
Gaucher	β-glucosidase acid (GBA)	glucosylceramide glucosylsphingosine	GBA intracellular trafficking endolysosomal trafficking, and autophagy	[130]
				[11]
GM1 gangliosidosis	β-galactosidase (GLB1)	GM1 ganglioside oligosaccharides	depletion of Ca^2+^ from the ER unfolded protein response apoptosis autophagy enhancement	[131]
				[132]
				[133]
				[134]
mucolipidosis type II/III	N-acetylglucosamine-1-phosphotransferase alpha/beta subunits (GNPTAB)	glycolipids	defective hydrolase targeting lysosomes impairment of constitutive autophagy	[135]
				[136]
mucolipidosis type IV	mucolipin-1 (MCOLN1)	mucopolysaccharides gangliosides	endosomal/lysosomal trafficking autophagy lysosomal exocytosis mTOR and TFEB signalling heavy metal homeostasis	[137]
mucopolysaccharidosis type I (Hurler Syndrome)	α-L-iduronidase (IDUA)	heparan sulfate dermatan sulfate	breakdown of lysosomal membranes elevation of organelle pH lysosomal hydrolase activity oxidative stress necrosis apoptosis	[138]
				[139]
				[140]
				[141]
mucopolysaccharidosis type II (Hunter syndrome)	iduronate-2-sulfatase (IDS)	heparan sulfate dermatan sulfate	growth factor and morphogen signalling dysregulation mitochondria functions apoptosis cell death autophagy enhancement	[142]
				[143]
				[18]
				[144]
				[145]
mucopolysaccharidosis type IIIA (Sanfilippo Syndrome A)	N-sulfoglucosamine sulfohydrolase (SGSH)	heparan sulfate	exocytosis impairment autophagy inhibition oxidative imbalance	[146]
				[4]
				[147]
mucopolysaccharidosis type IIIB (Sanfilippo Syndrome B)	α-N-acetylglucosaminidase (NAGLU)	heparan sulfate	block of autophagic flux	[12]
				[25]
				[148]
				[149]
mucopolysaccharidosis type IIIC (Sanfilippo Syndrome C)	heparan-α-glucosaminide N-acetiltransferase (HGSNAT)	heparan sulfate	impairment of normal protein folding mitochondrial pathology autophagy	[150]
				[151]
mucopolysaccharidosis type IIID (Sanfilippo Syndrome D)	N-acetylglucosamine-6-sulfatase (GNS)	heparan sulfate	lysosomal enzyme deficiency	[152]
				[153]
mucopolysaccharidosis type IVA (Morquio A syndrome)	N-acetylgalactosamine-sulfate sulfatase (GALNS)	keratan sulfate chondroitin 6-sulfate	pyruvate metabolism cytoskeleton organization vesicle trafficking mitochondria functions Golgilysosome interaction lysosomal membrane repair iron transport Ca^2+^ signalling	[154]
mucopolysaccharidosis type IVB (Morquio B syndrome)	β-galactosidase (GLB1)	keratan sulfate	lysosomal enzyme deficiency	[155]
mucopolysaccharidosis type VI (Maroteaux-Lamy syndrome)	arylsulfatase B (ARSB)	dermatan sulfate	autophagy impairment accumulation of polyubiquitinated proteins mitochondrial dysfunction apoptosis	[156]

				[157]
mucopolysaccharidosis type VII (Sly syndrome)	β-glucuronidase (GUSB)	dermatan sulfate heparan sulfate chondroitin 4-sulfate chondroitin 6-sulfate	expanded endocytic compartments accumulation of lipofuscin granules increased autophagosomes cathepsins upregulation	[158]
				[159]
mucopolysaccharidosis type IX (Natowicz syndrome)	hyaluronidase 1 (YAL1)	hyaluronic acid	ECM structure and function cell cycle cell motility RNA translation and splicing autophagy	[160]
				[161]
neuronal ceroid lipofuscinosis	CLN1, CLN2 CLN5, CLN10 and CLN13 CLN3, CLN7 and CLN12 CLN6 and CLN8 CLN4 and CLN14 CLN11	autofluorescent lipopigments	endolysosomal and autophagic pathways lysosomal acidification and endosome-lysosome and autophagosome–lysosome fusions	[162]
				[163]
				[164]
				[165]
				[166]
				[167]
				[168]
				[169]
				[170]
Niemann-Pick disease type A/B	sphingomyelin phosphodiesterase 1 acid lysosomal (SMPD1)	sphingomyelin cholesterol glycosphingolipids	defects in intracellular traffic of lipids autophagy	[171]
				[172]
				[173]
Niemann-Pick disease type C1/D	NPC intracellular cholesterol transporter 1 (NPC1)			[174]
Niemann-Pick disease type C2	NPC intracellular cholesterol transporter 2 (NPC2)			[175]
				[176]
				[177]
				[178]
				[71]
Pompe	α-glucosidase acid (GAA)	glycogen	lysosome-based signalling pathways defective autophagy metabolism	[179]
				[180]
Sandhoff	β-hexosaminidase (HEXB)	GM2 ganglioside	abnormal intracellular signalling cascades apoptosis	[181] [182]

The most characteristic histological feature of LSDs is the presence of enlarged lysosomes filled with undegraded materials [21,22,120,183]. Independently of its origin, the accumulation of non-degradable materials has a profound impact on organelle physiology, size, trafficking, secretion and overall degradative capacity [49,173,184,185]. For instance, sphingomyelin accumulation in Niemann-Pick cells blocks transient receptor potential (TRP) channel protein mucolipin-1 (TRPML1)- and calcium-dependent lysosomal functions, thereby affecting lysosomal physiological trafficking and positioning [173–176,186]. In Niemann-Pick type C (NPC), mutations in genes encoding the lysosomal transmembrane protein NPC1 cause aberrant cholesterol transport at the MCSs between the endolysosomal and the ER membranes leading to cholesterol accumulation in the lysosomal compartment [178]. Accumulated cholesterol triggers the formation of the Rab7-RILP-dynein–dynactin complex and lysosomal clustering at the MTOC area [71]. In addition, cholesterol buildup promotes the expansion of lysosome-mitochondria MCSs which leads to cholesterol transport and accumulation into mitochondria by the activity of the endolysosomal sterol-binding protein STARD3 [178]. Moreover, cholesterol accumulation triggers the recruitment of the multi-meric tethering complex HOPS on the LE/lysosome membranes to drive membrane fusion between LEs/lysosomes and autophagosomes, thereby contributing to the expansion of the degradative compartment and the consequent worsening of the pathology [93,94].

An altered localization of lysosomes, often clustered in the juxtanuclear area, seems to play a critical role also in the pathophysiology of Gaucher disease [130], neuronal ceroid lipofuscinosis type 3 (CLN3) [187] and mucolipidosis type IV (MLIV) [44]. In the mouse model of Gaucher disease, characterized by the accumulation of glucosylceramide and glucosylsphingosine inside the lysosomes due to the loss of the β-glucocerebrosidase enzyme, perinuclear localization of Limp-2 positive vesicles was detected prior to changes in gene expression and before any overt symptoms. This strongly suggests that impaired organelle trafficking might be the priming force in the pathogenesis of Gaucher disease [130,11]. In neuronal ceroid lipofuscinoses (NCL), the accumulation of autofluorescent ceroid lipopigments, subunit C of mitochondrial adenosine triphosphate (ATP) synthase or sphingolipid activator proteins A and D occurs in the lysosomes of most cells. NCL-causing altered proteins (CLN1–CLN14) include soluble lysosomal enzymes, polytopic membrane proteins localized in lysosomes or ER, or synaptic vesicle-associated proteins [188]. In particular, CLN3, the neuronal CLN3 transmembrane protein [189], has been shown to affect the steady state position and motility of endosomes/lysosomes, and late steps of the endocytic pathway through the interaction with Hook1 (Hook Microtubule-Tethering Protein 1), the microtubular motor protein complexes Rab7-RILP/ORP1L/dynein–dynactin, and kinesin-2 [187,190]. In addition, CLN3 may also affect retrograde transport from LEs to the Golgi complex. Both Btn1p and Btn2p, the yeast orthologues of CLN3-interacting with Hook1, are involved in the LE-to-Golgi transport of Yif1p, a member of a conserved family of transmembrane proteins that interact with Rab GTPases in yeast. However, while Btn1p likely regulates SNARE complex phosphorylation and assembly at the Golgi membranes, Btn2p localizes to vacuoles and associates there with retrieval components [191,192].

MLIV is a rare autosomal recessive LSD due to loss-of-function mutations in the *MCOLN1* gene encoding for mucolipin-1 (ML1), also known as TRPML1. ML1 is a vesicular Ca^2+^ release channel belonging to the TRP superfamily [193] and has been associated with endosomal and autophagosomal trafficking, abnormal regulation of lysosomal exocytosis, changes in the mTORC1/TFEB signalling axis and dysregulation of heavy metal homeostasis [137]. The channel regulates Ca^2+^ transport across membranes towards the lysosomes, Ca^2+^-dependent fusion between lysosomes and PM during the exocytotic stage of membrane trafficking, and pathophysiological processes related to lysosomal aggregation, proteolysis and storage [194,195]. Finally, ML1 dysfunction has been associated with autophagy impairment [196]. Indeed, defective autophagy characterized by incomplete degradation of autophagosomes and their accumulation in the cytoplasm has been observed in both human fibroblasts from patients affected by MLIV and neuronal cultures from the mouse model of the disease [137].

Autophagy impairment has also been demonstrated in many mucopolysaccharidosis subtypes [18,197,198], such as MPS II [18], MPS IIIA [147], MPS IIIB [12], MPS IIIC [151] and in MPS VI [157]. The dysregulation of mTORC1 signalling and autophagy affects ECM formation, skeletal development and bone growth in some MPSs [199,200]. In a mouse model of MPS II, using antibodies against subunit C of mitochondrial ATP synthetase and p62, immunohistological changes showing increased autophagosome vacuolation were observed in neurons, microglia and pericytes of mice suggesting a block of autophagosome–lysosome fusion [145]. In a *Drosophila* model of MPS IIIA, the block of autophagy has been shown to represent an important pathogenetic factor for neurodegeneration [4]. Valvular abnormalities and cardiac failure have been associated with impaired lysosomal autophagic flux in the mouse model of MPS IIIB [12]. An impairment of neuronal autophagosome–lysosome fusion and mitophagy was reported in the mouse model of MPS IIIC which would account for the progressive accumulation of gangliosides, aggregates of subunit C of mitochondrial ATP synthase and deformed and dysfunctional mitochondria [151,201]. Impairment of autophagy, accumulation of polyubiquitinated proteins and mitochondrial dysfunction were observed in fibroblasts derived from MPS VI patients and the rat model of the disease [156]. A regulatory activity on autophagy has been also associated with hyaluronidase 1, which is the defective enzyme in MPS IX [161].

In LSDs, primary substrate accumulation triggers a cascade of events leading to the accumulation of either cytosolic or luminal secondary substrates which generates defects in lysosomal reformation and the autophagic flux due to the impairment of the autophagosome–lysosome fusion [20,173, 202,203]. Indeed, modifications of the lysosomal-autophagy mechanisms have been ascertained in many other LSDs [164], including Pompe disease [179], sphingolipidoses such as Gaucher disease [204], Fabry disease [205,206], and NPC [207,208], mucolipidosis II [135] and IV subtypes [209], Danon disease [126], and some NCLs [164,210]. In Anderson–Fabry disease, the accumulation of sphingolipid substrates in lysosomes inhibits autophagosome–lysosome fusion and disrupts the mTOR activation/inactivation cycle, interfering with the mTOR-mediated control of mitochondrial metabolism [129]. Disturbed autophagy and activated microglia have been described in a GM1 gangliosidosis mutant mouse model [133]. Following glycogen accumulation in lysosomes, the dysregulation of AMPK and mTORC1 signalling pathways, defective autophagy, muscle proteostasis, oxidative stress and dysregulation of the major metabolic pathways have been demonstrated in Pompe disease [180]. In the mouse model of Danon disease, LAMP2-deficient hepatocytes show accumulation of early autophagic vacuoles, mistargeting of lysosomal enzymes including LAMP1, improper cathepsin D processing, abnormal retention of mannose-6-phosphate receptors in autophagic vacuoles, reduction of the degradation of long-lived proteins and resistance to autophagy-dependent protein breakdown during starvation [126]*.* Impairment of autophagy has been implicated as also contributing to the pathogenesis of NPC disease. Indeed, NPC1 deficiency results in the marked accumulation of autophagosomes in neurons of *Npc1*^−/−^ mice and primary fibroblasts from patients. However, the disease is also associated with diminished autophagic flux [172]. In the brain of mucolipidosis type II knockout mice, the accumulation of fucosylated *N*-glycans, GM2 and GM3 gangliosides, cholesterol, and bis(monoacylglycerol)phosphate was accompanied by an increased neuronal level of the microtubule-associated protein 1 light chain 3 and the formation of p62-positive neuronal aggregates indicating an impairment of constitutive autophagy [135]. Deregulation of autophagy has also been demonstrated in various NCL mouse models. A block of autophagic flux due to the accumulation of autophagosomes and autophagic substrates associated with impaired lysosomal functions in CLN2, CLN5, CLN6 and CLN7 knockout mice has been reported [165–167]. Defective autophagosome maturation has been detected in both the CLN3 mouse model and in fibroblasts derived from patients as well as in neuronal cells derived from patient-specific induced pluripotent stem cells [164,168,169,211].

Interestingly, a lack of autophagy completion in LSDs leads to the persistence of ubiquitinated and aggregate-prone polypeptides in the cytoplasm, including p62/SQSTM1, α-synuclein and Huntingtin protein [156,184,212,213]. Moreover, α-synuclein itself contributes to neurodegeneration by reducing the efficiency of autophagosome formation [214] and is also the main component of Lewy bodies that are usually elevated in Parkinson's disease and other forms of dementia. Thus, the diminished quality control of cytosolic proteins can also contribute to LSD pathology [215]. In particular, α-synuclein accumulation has been suggested to promote neurotoxicity in Gaucher [216], Niemann-Pick [217] and Krabbe diseases [218]. In a mouse model of sialidosis, the deficiency of the lysosomal sialidase NEU1 (neuraminidase 1) leads to the accumulation of an oversialylated amyloid precursor protein in the lysosomes and extracellular release of amyloid *β* (*αβ*) peptides by excessive lysosomal exocytosis [219]. In a murine model of GM1 gangliosidosis, swollen neurons showed intra-lysosomal storage of lipids extending into axons and amyloid precursor protein-positive spheroids. Furthermore, axons exhibited a higher kinesin and lower dynein immunoreactivity compared to wild-type controls [220]. Deposition of α-synuclein together with other amyloidogenic proteins has been observed in diverse types of MPSs characterized by a severe neurological phenotype [215,221]. Moreover, lysosomal proteolysis inhibition has also been found to disrupt axonal transport of LEs, lysosomes and autolysosomes in neurons, resulting in their accumulation in dystrophic axonal swellings characteristic of Alzheimer's disease [222,223].

## 6. Conclusion

(1) The subcellular positioning of lysosomes between endocytic/phagocytic and autophagic processes allows them to integrate extracellular and intracellular stimuli, and, accordingly, control cellular adaptation. Although the lysosomal compartment was long considered a waste disposal and recycling centre, emerging studies support its new role as a platform to initiate, organize and coordinate diverse signalling events.
(2) The most widely used mechanism for intracellular transport involves molecular motor proteins that carry many different cargos. Several types of macromolecular machineries control bidirectional lysosomal motility on microtubule tracks, and their specificity for each membrane-enclosed cargo depends on the precise molecular composition, including adaptor proteins and lipids, facing the cytosolic side of the membranes.
(3) The organelles can establish MCSs via protein tethers composed of multiple classes of proteins. Many of these proteins in the tethers have comparable properties and/or functions. For example, tethers can include structural proteins (some of them contain motifs able to bridge two closed membranes) and functional proteins (such as ion channels and lipid transfer proteins), as well as putative regulatory proteins.
(4) In LSDs, the undegraded substrate storage within the lysosomes has a profound impact on organelle size, trafficking, secretion and physiological degradative activity. Growing evidence demonstrate distinctive disturbances of lysosomes in LSDs resulting in unique patterns of auto/endolysosomal mis-trafficking.
(5) Dysregulation of lysosomal functions plays a key role not only in LSDs but also in a broad variety of neurodegenerative diseases, cardiovascular diseases, metabolic disorders and cancer. The new vision of the central role of the lysosome in multiple cellular functions such as energy metabolism, cell proliferation and differentiation, immunity, and cell death has significantly advanced our knowledge of the pathophysiology of lysosomal-related diseases, paving the way for the development of novel therapeutic strategies [224].

## Data Availability

This article has no additional data.
